# Advances in endogenous RNA pull-down: A straightforward dextran sulfate-based method enhancing RNA recovery

**DOI:** 10.3389/fmolb.2022.1004746

**Published:** 2022-10-19

**Authors:** Fabio Desideri, Eleonora D’Ambra, Pietro Laneve, Monica Ballarino

**Affiliations:** ^1^ Center for Life Nano- & Neuro-Science of Istituto Italiano di Tecnologia (IIT), Rome, Italy; ^2^ Institute of Molecular Biology and Pathology, National Research Council, Rome, Italy; ^3^ Department of Biology and Biotechnologies “Charles Darwin”, Sapienza University of Rome, Rome, Italy

**Keywords:** RNA pull-down, RNA/RNA interactions, lncRNA, microRNA, Dextran sulfate sodium salt

## Abstract

Detecting RNA/RNA interactions in the context of a given cellular system is crucial to gain insights into the molecular mechanisms that stand beneath each specific RNA molecule. When it comes to non-protein coding RNA (ncRNAs), and especially to long noncoding RNAs (lncRNAs), the reliability of the RNA purification is dramatically dependent on their abundance. Exogenous methods, in which lncRNAs are *in vitro* transcribed and incubated with protein extracts or overexpressed by cell transfection, have been extensively used to overcome the problem of abundance. However, although useful to study the contribution of single RNA sub-modules to RNA/protein interactions, these exogenous practices might fail in revealing biologically meaningful contacts occurring *in vivo* and risk to generate non-physiological artifacts. Therefore, endogenous methods must be preferred, especially for the initial identification of partners specifically interacting with elected RNAs. Here, we apply an endogenous RNA pull-down to lncMN2-203, a neuron-specific lncRNA contributing to the robustness of motor neurons specification, through the interaction with miRNA-466i-5p. We show that both the yield of lncMN2-203 recovery and the specificity of its interaction with the miRNA dramatically increase in the presence of Dextran Sulfate Sodium (DSS) salt. This new set-up may represent a powerful means for improving the study of RNA-RNA interactions of biological significance, especially for those lncRNAs whose role as microRNA (miRNA) sponges or regulators of mRNA stability was demonstrated.

## Introduction

Since the first characterization of RNA, DNA and protein chemical composition, it has becoming increasingly clear the need to carve the interactions that these macromolecules establish in living cells to execute their functions (Matthews, 1988; Moore, 2005). On this line, protein-centric methodologies for the antibody-mediated detection of specific peptide/nucleic acid contacts (Phizicky and Fields 1995), have greatly impacted molecular biology research. Along with the discovery of several classes of non-protein-coding RNAs, the need to study their functions prompt to develop increasingly refined technologies for characterizing their physical association with individual proteins (as reviewed in Cipriano and Ballarino, 2018). The native RNA immunoprecipitation (RIP) (Lerner and Steitz 1979) represented the first method developed for this purpose, which was followed in the early 2000s by the development of UV-crosslinking and immunoprecipitation (CLIP) assays and subsequent CLIP-based methods (Ule et al., 2003; Lee and Ule 2018; Hafner et al., 2021). Over the years these approaches were gradually flanked by the development of complementary RNA-centric methods, in which the putative interactors of a given RNA are specifically purified by using antisense DNA probes as baits (McHugh et al., 2015; Ramanathan et al., 2019).

Among the RNA-centric approaches, the methodologies allowing to detect RNA/RNA contacts train great attention, also in light of the ever-increasing importance of base-complementarity between transcripts, both in a mechanistic and in a regulative perspective (Helwak et al., 2013; Engreitz et al., 2014; Guil and Esteller, 2015). Although these strategies already contribute in a significant manner to functional/descriptive studies of RNA-RNA and protein interactions, continuous adjustments are required to adapt the experimental protocols to specific case studies. The latter observation largely applies to long noncoding RNAs (lncRNAs) which, especially in the last three decades, have been shown to greatly contribute to regulate gene expression in the nucleus and cytoplasm (Li and Chang, 2014; Herman et al., 2022). Consequently, aberrant lncRNA expression has been implicated in a variety of human diseases, including cancer, cardiovascular and neurological disorders (Fatima et al., 2015; Lekka and Hall, 2018; Ni et al., 2022).

LncRNAs represent an heterogenous class of non-protein coding molecules arbitrarily defined as transcripts longer than 200 nucleotides, which regulate gene expression through transcriptional as well as post-transcriptional mechanisms (Yao et al., 2019; Statello et al., 2021). Many of them are not constitutively active but exert regulatory roles, which makes their study particularly challenging. In fact, their low abundance at the steady-state level, their restricted expression to specific cell subtypes or developmental windows and their modest evolutionary conservation, often makes the lncRNA-mediated gene regulation extremely circumscribed and hard-to-be unraveled (Fatica and Bozzoni 2014; Kopp and Mendell, 2018).

From a mechanistic standpoint, lncRNAs function through the interaction with other biomolecules (Ferrè et al., 2016), and several examples suggest a crucial role for local lncRNA-RNA contacts at the root of their activities (Gong and Maquat, 2011; Carrieri et al., 2012; Kretz et al., 2013; Martone et al., 2020). Overall, these observations suggest that practices which aim to ameliorate the identification of bound partners from complex cellular extracts represent a critical step to clarify noncoding RNA-mediated cellular activities. Several databases and *in silico* tools were developed for the prediction of lncRNA functions based on lncRNA-RNA interactions (Bellucci et al., 2011; Fukunaga, and Hamada, 2017; Gong et al., 2018; Fukunaga et al., 2019). In parallel, experimental procedures have evolved to globally enhance the reliability and efficacy in detecting RNA-RNA interplays (Cai et al., 2020).

Hereinafter, we propose a strategy which represents a variation on the theme of the standard RNA pull-down (PD), with the innovative use of the Dextran Sulphate Sodium (DSS) salt as a hygroscopic chemical additive. By testing the procedure to the motoneuron-expressed lncRNA lncMN2-203 (Biscarini et al., 2018; Carvelli et al., 2022), we found that DSS 1) greatly improves the purification of lncMN2-203, as compared to previous analysis and, importantly, 2) facilitates the identification of its RNA binding partner, the microRNA miRNA-466i-5p. These results promise to upgrade RNA functional analyses in a straightforward and effective manner.

## Materials and equipment

### Oligonucleotide probe design and sequences

A number of 15 oligonucleotide probes, 90-nucleotides long and carrying a 5’-biotin modification, were designed as in McHugh et al., 2015 to cover the lncMN2-203 sequence. U1 snRNA Probes (Desideri et al., 2020) were used as a control.

## Buffer composition

### Lysis buffer

Tris-HCl pH 7.5 50 mM, NaCl 150 mM, MgCl2 3 mM, NP40 0.5%, EDTA 2 mM. Add fresh DTT 1 mM, 1× PIC, and RNase Inhibitors (0.2 U/μl).

### Hybridization buffer (HB)

Tris-HCl pH 7.5 100 mM, NaCl 300 mM, MgCl2 1 mM, SDS 0.2%, Formamide 15%, NP40 0.5%, EDTA 10 mM. Add fresh DTT 1 mM, 1× PIC, and RNase inhibitors (0.2 U/μl).

## Reagent list

1) Streptavidin Magnesphere paramagnetic beads—Promega.
2) Dextran Sulfate Sodium (DSS) salt—SigmaAldrich
3) cOmplete™, Mini, EDTA-free Protease Inhibitor Cocktail (PIC)—ROCHE.
4) RNase Inhibitor: RiboLock—ThermoFisher Scientific.
5) Tris, NaCl, MgCl2, SDS, Formamide, NP40, EDTA and Dithiothreitol (DTT)—SigmaAldrich.
6) TRI-Reagent—Zymo Research.
7) Direct-zol RNA Miniprep Kit and DNase—Zymo Research.
8) Superscript VILO™ cDNA Synthesis Kit—ThermoFisher Scientific.
9) SYBR Green Power-UP - ThermoFisher Scientific
10) miScript II RT Kit—QIAGEN
11) miScript SYBR Green—QIAGEN.

## Experimental procedure

mESCs carrying the Hb9:GFP transgene (Wichterle et al., 2002) are differentiated to motoneurogenesis through embryoid bodies (EB) formation as previously described (Wichterle and Peljto, 2008). Embryoid bodies at day 6 of differentiation (EB6) are harvested in PBS and centrifuged at 400 x g for 5 min, before proceeding as follows:

1) Gently resuspend cell pellets from EB6 in lysis buffer supplemented with proteases (1X PIC- ROCHE) and RNase inhibitors (0.2 U/μl—Thermo Fisher Scientific). Live, freshly isolated, not frozen cells should be preferred.
2) Incubate the cell suspension first on ice (10 min) and then on a rotating wheel for 10 min at 4°C. Centrifuge the cell suspension at 15,000 × g at 4°C for 15 min and collect the supernatant, which represents the total cellular extract, into new tubes. Quantify the extract by protein determination and dilute 1:2 in Hybridization Buffer (HB). From the diluted extracts, collect 1 mg of material for each PD condition and 0.1 mg for the Input (i.e., 10% of the PD sample). Typically, 10 millions of freshly lysed EB6 yield up to 1 mg of total extract. CRITICAL STEP: since DSS can interfere with enzymatic reactions, collect Input before DSS addition to the EB6 total extract or use specific RNA purification methods to remove possible salt contaminants. A final volume of 0.9 ml in a 1.5 ml vial tube is the ideal condition for hybridization.
3) Add DSS salt (SigmaAldrich) to each PD sample at a final concentration of 1% or 2.5%. CRITICAL STEP: DSS can be very viscous when undiluted, be careful to pipette the required volume. Cutting the tip of the pipette could help.
4) Dilute 100 pmol of 5′-biotinylated (90-mer long) antisense DNA oligonucleotide probes specifically targeting lnc-MN2 or U1snRNA to a final volume of 50 μL with HB. Heat-denature the probes for 3 min at 80°C and directly add to each sample (MN2 PD, U1 PD).
5) Mix the specific probes with the cellular extract and incubate at 4°C for, at least, 4 h. Incubate Input and PD samples in parallel. Before concluding the incubation, gently wash 0.1 ml of Streptavidin paramagnetic beads (Promega)/sample with HB on a magnetic rack (Millipore). Repeat this step. CRITICAL STEP: prior to pipette the needed amount of beads, gently flick the bottom of the tube until the particles are completely dispersed.
6) Resuspend the Streptavidin paramagnetic beads in 0.1 ml of fresh HB and then add to each PD sample.
7) Incubate beads/extract/probe for 1 h at RT (20–25°C). On a magnetic rack, remove the supernatant from the beads and discard as it contains the unbound material. Keep the beads for steps 8–9!
8) Carefully wash the beads 4 times (3 min each) on a rotating wheel with HB at RT (20–25°C). After each wash, recover the beads on a magnetic rack.
9) For RNA extraction, add Trizol (TRI Reagent- Zymo Research) directly on the washed beads and vortex thoroughly for 2 min to detach the probes. Place back the samples on the magnetic rack and collect the supernatant in a new 1.5 ml tube. CRITICAL STEP: the supernatant contains RNA. Keep it for RNA extraction!
10) Purify the RNA with Direct-zol RNA Miniprep Kit (Zymo Research), according to manufacturer’s instructions and treat with DNase at RT (20–25°C). Elute RNA in 30 μL of Elution Buffer (Zymo Research) or DNase/RNase-Free Water. *OK to store RNA at −20°C at this point.
11) For long RNA transcript quantification, perform reverse transcription with SuperScript™ VILO™ cDNA Synthesis Kit (ThermoFisher Scientific), according to manufacturer’s instructions and qRT-PCR with SYBR Green Power-UP (ThermoFisher Scientific). For miRNA quantification, perform reverse transcription with miScript II RT Kit (QIAGEN) and qRT-PCR with miScript SYBR Green (QIAGEN). 250–500 μg of RNA for each reverse transcription reaction is the ideal amount.

## Results

The abovementioned protocol is thought to optimize the performance of the classical endogenous RNA PD procedures, with the aim to increase the recovery of the targeted lncRNA and, consequently, the specific enrichment of its co-precipitated RNA interactors.

**FIGURE 1:**
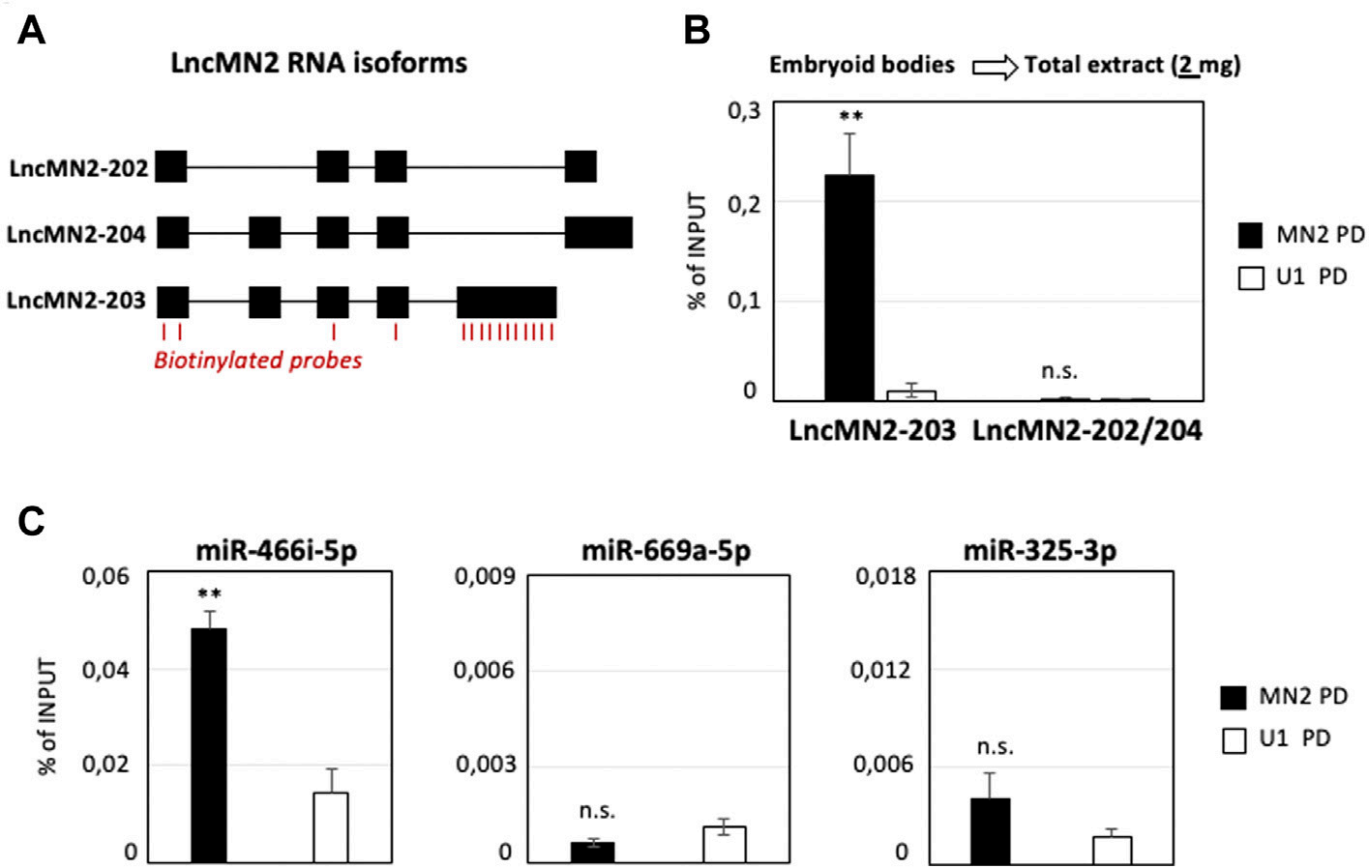
LncMN2-203 RNA pull-down in mESCs-derived embryoid bodies. **(A)** Block-and-line scheme representing the structure of the three lncMN2 RNA isoforms (Carvelli et al., 2022). Exon (black blocks)/intron (thin lines). The position of the antisense probes used for the RNA pull-down experiments is highlighted in red. **(B)** Quantification by qRT-PCR of lncMN2-203 and lncMN2-202/204 recoveries from lncMN2-203 (MN2) or control (U1 snRNA) RNA pull-down experiments. A total of 2 mg cell extracts prepared from differentiated EB6 were used for each reaction. LncRNA enrichments are expressed as Input percentage (%). Error bars represent SEM. n = 3 biological replicates. **(C)** Quantification by qRT-PCR of miR-466i-5p (Left), miR-669a-5p (Middle) and miR-325-3p (Right) recoveries from lncMN2-203 (MN2) or control (U1 snRNA) pull-down experiments. MiRNA enrichments are expressed as Input percentage (%). Error bars represent SEM. *n* = 3 biological replicates. Data information: **p ≤ 0.01, n. s.> 0.05 (two-tailed, unpaired Student’s t-test).

The efficacy of the procedure was tested on lncMN2-203 (Figure 1A), a motor neuron (MN) specific lncRNA (Biscarini et al., 2018) recently found to control the specification of murine MNs by acting as a miRNA sponge (Carvelli et al., 2022). By using the same amount of total cell extract (2 mg of Input) previously used for lncMN2-203 precipitation (Carvelli et al., 2022), we reproduced its significant enrichment as compared to U1 snRNA PD, used as a control (Figure 1B). In accordance with the earlier analyses, the recovery of the other lncRNA isoforms (lncMN2-202/204) was almost null, in both the specific and control samples. In this setting, we also checked for miRNAs known to co-precipitate with lncMN2-203 and we confirmed that miR-466i-5p was significantly enriched in the MN2 as compared to the U1 PD (Figure 1C, left panel). MiR-669a-5p and miR-325-3p, used as negative controls, were almost undetectable in both the conditions (Figure 1C, middle and right panels), in line with their known inability to interact with the lncRNA (Carvelli et al., 2022).

Building upon these results, we wondered whether we could improve the cost-effectiveness of the procedure without reducing the performances. The evidence that lncRNA expression is often restricted to specific cell sub-populations causes not negligible concentration constrains (Goff et al., 2015). This is even more evident when the total cellular extracts are not prepared from FACS-sorted cells but rather from mixed cell populations, where the lncRNA average expression might be diluted. These aspects, that severely risk to impact the performance of the endogenous PD approaches, make lncMN2-203 as the most suitable candidate for testing the protocol. In fact, previously performed single-cell RNA-sequencing (scRNA-seq) showed that lncMN2-203 expression is restricted to a specific subpopulation of EB6 cells, namely the late MNs (Carvelli et al., 2022).

**FIGURE 2:**
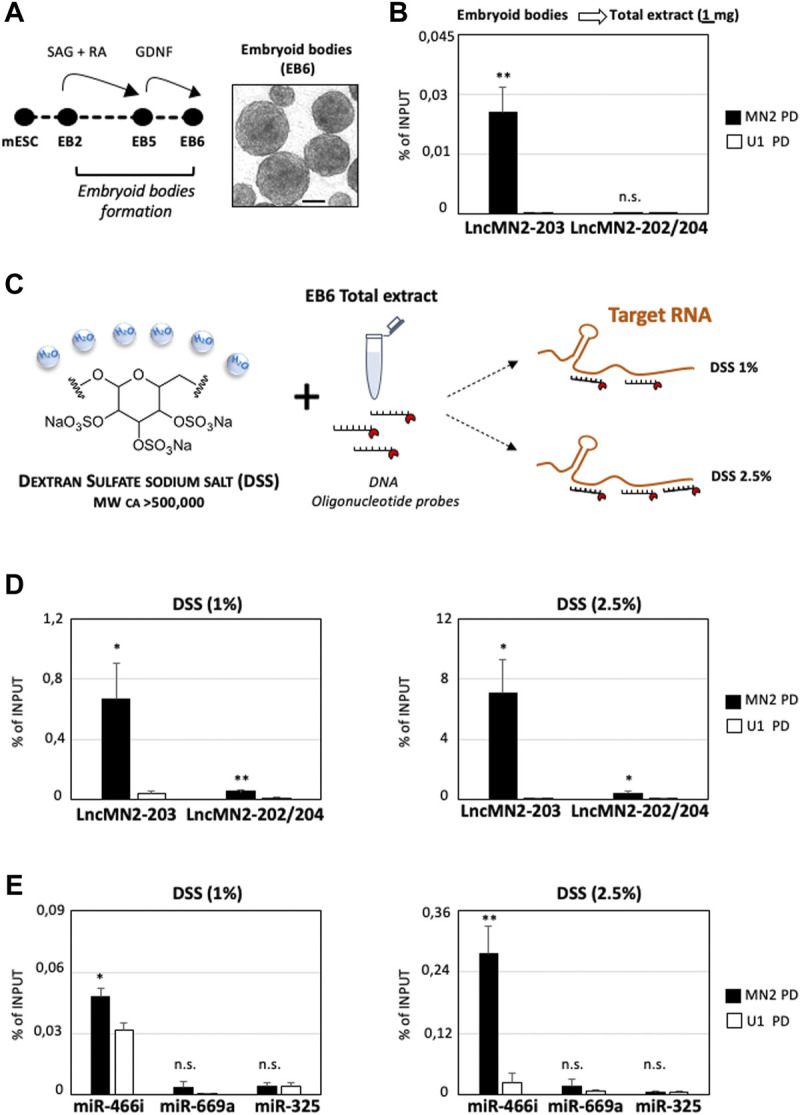
Dextran Sulfate Sodium salt increases the enrichment of LncMn2-203 and its miRNA interactor. **(A)** Left: Schematic representation of mESC-derived embryoid bodies differentiation (Capauto et al., 2018; D’Ambra et al., 2021). A representative field of EBs at day 6 of differentiation (EB6) is shown aside. Scale bar: 100 μm. SAG = Smoothened Agonist; RA = Retinoic Acid; GDNF = Glial cell line-derived neurotrophic factor. **(B)** Quantification by qRT-PCR of lncMN2-203 and lncMN2-202/204 recovery from lncMN2-203 (MN2) or control (U1 snRNA) pull-down experiments. A total of 1 mg of cell extracts prepared from differentiated EB6 were used for each reaction. LncRNA enrichments are expressed as Input percentage (%). Error bars represent SEM. *n* = 3 biological replicates. **(C)** Scheme of the Dextran Sulfate sodium salt (DSS) chemical structure and hygroscopic properties. By sequestering H20 molecules, the DSS salt increases the local concentration of DNA antisense probes targeting nucleic acid. **(D)** Quantification by qRT-PCR of lncMN2-203 and lncMN2-202/204 recoveries from lncMN2-203 (MN2) or control (U1 snRNA) RNA pull-down experiments. Different doses of DSS, 1% (Left) or 2.5% (Right), were used for each reaction. LncRNA enrichments are expressed as Input percentage (%). Error bars represent SEM. *n* = 3 biological replicates. **(E)** Quantification by qRT-PCR of miR-466i-5p, miR-669a-5p and miR-325-3p recoveries from lncMN2-203 (MN2) or control (U1 snRNA) RNA pull-down experiments. Different doses of DSS, 1% (Left) or 2.5% (Right), were used for each reaction. MiRNA enrichments are expressed as Input percentage (%). Error bars represent SEM. *n* = 3 biological replicates. Data information: *p ≤ 0.05, **p ≤ 0.01, n. s.> 0.05 (two-tailed, unpaired Student’s t-test).

With the aim to reduce unnecessary costs, lncMN2-203 PD was repeated by halving the Input used in our original set-up (Figure 1A) to 1 mg of total lysate. Such a scale-down can be particularly useful when the targeted RNA is expressed in model systems whose maintenance and handling is costly and labor intensive. The mouse embryonic stem cells used for the characterization of lncMN2-203 perfectly fit in this example, as they require multiple passages for cell expansion and differentiation towards MNs, through the formation of EB (Figure 2A). In these new conditions, while the recovery of the lncRNA remained significant compared to the U1 control, the lncMN2-203 yield, calculated as percentage of Input, was about ten-fold lower than previously obtained (Figure 2B and Supplementary Figure S1A). More importantly, the diminished RNA recovery was not sufficient to co-precipitate the lncMN2-203 interactor, miRNA-466i-5p (Supplementary Figure 1A).

Exploring further resolutive options, we considered to use DSS salt, based on its well-known ability to accelerate probes-to-target hybridization (Lederman, et al., 1981), increasing fluorescent signals (Van Gijlswijk, et al., 1996) and reducing background (Singh and Jones, 1984). Together, these properties make the DSS particularly useful in RNA Fluorescence *In Situ* Hybridization (RNA-FISH) experiments for the visualization of both mRNA (Singer and Ward, 1982) and other transcripts which are less abundant, as with most lncRNAs (Ballarino et al., 2018; Santini et al., 2021). This feature is due to the chemical composition of this polysaccharide containing 17–20% sulfur that, being a highly water-soluble natural polymer, sequesters H2O molecules and enhances the effective concentration of DNA probes available for nucleic acid targeting (Figure 2C and Wetmur, 1975). Normally, the working concentration of DSS on membrane-immobilized or cell-fixed nucleic acids is ∼10% of the final reaction volume (Wahl et al., 1979). At these concentrations of DSS, the solution appears viscous, and this could hamper the homogeneous mixing of RNA/protein extracts with the specific antisense oligonucleotide probes, which is crucial for the success of the PD assay. For this reason, the RNA PD was repeated by keeping constant the extract amount (1 mg), which was incubated with two different concentrations of DSS, specifically 1% and 2.5% of the final reaction volume (Figure 2C). Interestingly, in the two conditions we obtained a ∼25 and ∼280 -fold enrichment of lncMN2-203 respectively, measured as percentage of Input, as compared to the PD performed without DSS (DSS minus, Figure 2D and Supplementary Figure 1B). This dramatic enhancement in lncMN2-203 purification was accompanied by a slightly significant (but much lower respect to lncMN2-203) enrichment of the lncMN2-202/204 isoforms, as compared to the control (Figure 2D and Supplementary Figure 1B). This may be explained by the partial overlap between lncMN2-203 and lncMN2-202/204 sequence isoforms, together with the fact that a fraction (4 out of 15) of the capturing oligonucleotides targets the lncMN2 shared exons (Table 1).

**TABLE 1:** List of biotinylated probes used for LncMN2 RNA Pull down.

LncMN2. PD1	AAC​TTC​TGG​CCA​TTT​TCA​ACC​CAT​TTG​CTC​CAG​TTC​ACA​GCA​CTC​ACG​CAG​AAG​TAT​GGC​ACT​GAG​GGG​CT CAG​GAT​ACC​TCA​GGA​ATG​A
LncMN2. PD2	ATC​TTC​CTG​GAT​TTA​CCG​ACC​TCA​GGC​TCC​AGT​TCT​GCA​TAA​TTA​GCT​TAA​ACT​GGC​TCA​AAT​GGA​TTT​TAA​CTG​GTC​AGA​AAT​TCA​ATT
LncMN2. PD3	TCT​TGG​AGC​CTT​GGT​TTT​CTC​ATC​ATA​TCA​CAA​AGC​CTC​CAG​TCA​CCA​CAG​GGC​CAG​GGT​GAA​GTC​AAG​GAA​GAA​GTG​AAG​CTG​GAA​TCC
LncMN2. PD4	ACT​CCG​TGA​AGG​TGC​TGG​CTC​TTA​GGC​CAC​TTA​ATA​GCT​GCA​TTC​TAG​GGA​GCA​GCA​GGA​TAA​CAG​GGA​AAC​CAG​AAG​CTG​ATG​ACT​GGC
LncMN2. PD5	AGA​AGG​CCT​CCA​ACA​CAA​GCA​TCA​GCC​TCC​TTG​AGA​CTA​TAT​AGA​TCA​CTA​TTG​CTG​TTA​GTT​CAA​GCA​TTG​GGA​ATT​TCC​ATA​GGC​TGA
LncMN2. PD6	AAG​AAT​GAT​GGA​GTT​TCT​GCT​TTT​ATG​GTT​CAT​TCC​TTG​TGT​ACA​GCA​GAG​GAA​AAA​GTG​TTT​ATA​AGG​CCA​GAT​GGA​TAT​GGA​AGA​TGC
LncMN2. PD7	TCA​TCC​CAG​AGC​CAG​CAG​AAC​CCA​CTG​GCT​CAA​CTG​CAC​AAC​AAT​TTT​CAA​CAG​TCC​ACA​TAT​TAA​AGG​GCT​TTT​CAA​CAA​TGT​GGT​TCT
LncMN2. PD8	CTA​TAC​ATC​AGC​TAC​AAT​CAT​GTA​CTG​GCA​CTG​GGC​TAA​AGA​CCA​TGT​GAC​TTT​ATC​TTC​CTT​CAA​TAT​GGT​ATT​AAT​TTT​CAC​CAA​GAA
LncMN2. PD9	GCC​CAG​GCA​GAG​TTG​GAT​GGT​GGA​GGA​AGT​CCA​CAA​CCC​TAG​CCT​AAA​AAG​CAG​TGT​GCT​AGT​GGT​GCA​GAT​CAA​CTC​TAC​ATT​CCT​ATC
LncMN2. PD10	TGA​TCC​CTT​TCT​TAT​GGT​GAG​CTC​AGT​CCT​CTG​AGG​ACT​TCT​GTT​GTT​GCT​GTT​AAT​TAA​GAC​TGA​CAT​AAG​GAG​ACA​AGC​AGA​CAT​TGA
LncMN2. PD11	TTA​TGT​GTG​ACT​GTG​TGT​CCC​ACA​CTC​AGT​GAG​TAA​CTT​TGA​ACC​AAG​GAT​AAG​AAT​TGA​GTT​GGA​GCC​TAG​GAC​TTG​GGG​AAG​AAG​GCT
LncMN2. PD12	TAA​ATA​TAA​ACA​TCC​ACA​TTA​AAA​TCA​CCT​TAG​TCA​GCT​ATG​AGT​GAA​GAA​TAT​GTA​TCC​TAC​ACA​GTT​CTT​TGC​TAG​ATA​TTT​TAG​CTC
LncMN2. PD13	CCC​TCC​CAG​AAT​GAT​CAA​CAG​ATC​CTG​TAT​TAT​AAG​TTC​TAA​GAG​TGT​CTA​CTT​TTG​CAA​CAG​CTC​TGA​TCC​ATT​TGT​GGT​CAT​AGT​CAT
LncMN2. PD14	CTC​AGT​TGG​GAC​CCA​ATG​ATG​TTA​ACC​TTA​GTC​TTG​TGC​TTG​TTG​TAT​TTC​TCC​ACT​ATG​AGC​TGA​CTT​TGC​CTC​TTA​TTC​TGG​ACT​CCA
LncMN2. PD15	AGA​TAG​AGA​TCC​GTT​TTT​CTC​TTC​TTC​TCC​ATC​ATT​TTA​CCA​TGT​TTT​TTC​CAT​TGT​CTT​TAA​ACT​TGA​AGC​AGA​ATC​TTT​ATT​TTA​ATG

Encouraged by these results, we then checked for lncMN2-203 miRNA interactors in the 1% and 2.5% DSS PD samples. Interestingly, we found that both the percentage and the specificity of miRNA purification responded to the efficiency of lncMN2-203 PD, as miR-466i-5p enrichment was significantly higher in the 2.5% than in the 1% DSS sample (Figure 2E and Supplementary Figure 1B). Importantly, in none of the two conditions any significant enrichment was found for the negative controls, the miRNAs miR-669a-5p and miR-325-3p, which is in line with the specificity of the antisense oligonucleotide probes, even in the presence of DSS (Figure 2E and Supplementary Figure 1B).

**FIGURE 3:**
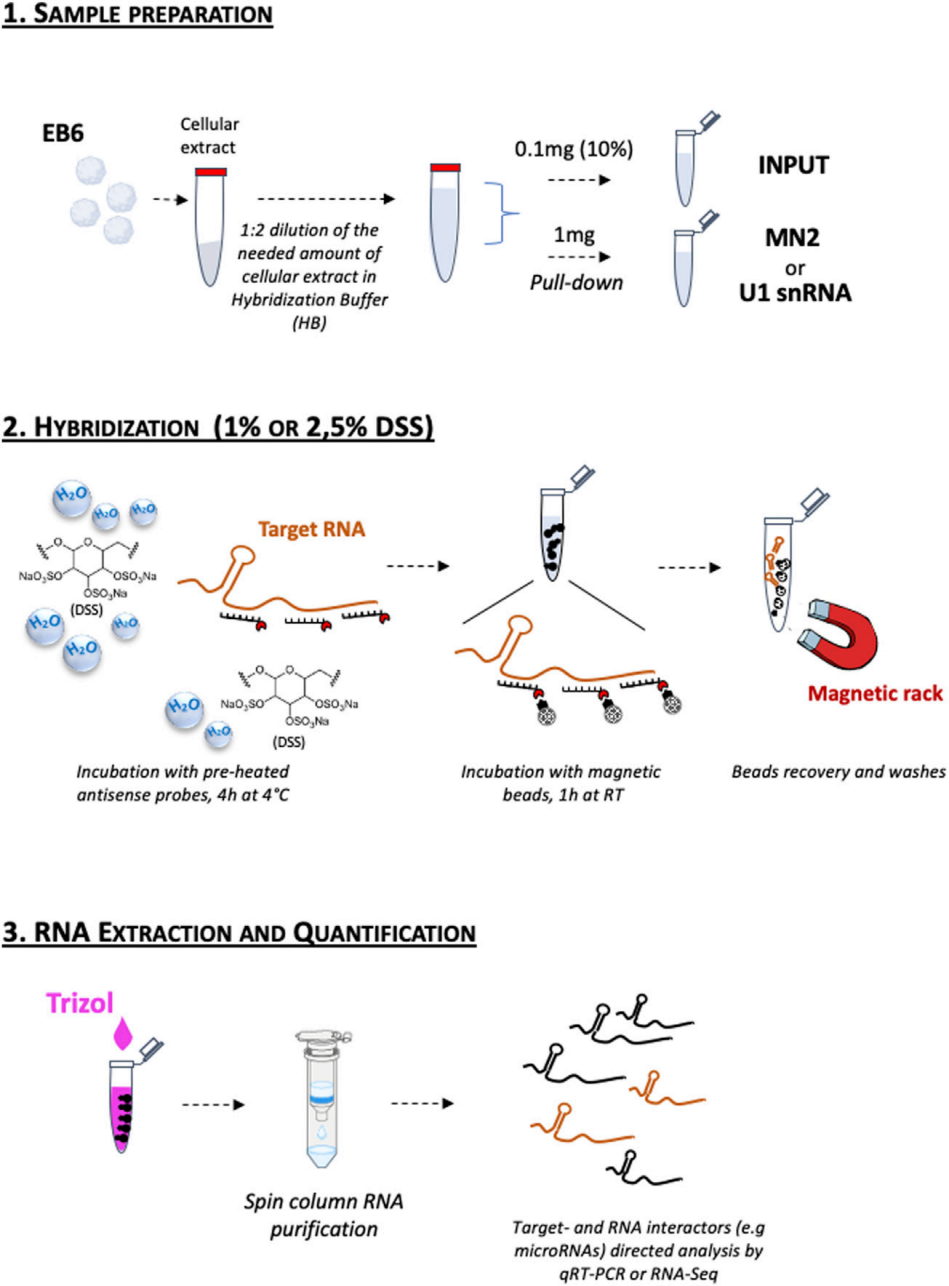
Schematic representation of the DSS-based RNA pull-down assay workflow. 1. Sample preparation; 2. Hybridization; 3. RNA Extraction and Quantification. The detailed protocol is reported in the Experimental Procedure section.

It was previously reported that DSS inhibits the activity of reverse transcriptase enzymes and the amplification by PCR (Kerr et al., 2012; Viennois et al., 2013; Juritsch and Moreau, 2019). However, the DSS-treated PD samples were normally PCR-amplified, likely because the repeated washing steps which precede the RNA extraction (see Experimental Procedure) remove any DSS residue potentially interfering with the RNA analysis. For these reasons, we recommend collecting the Input (EB6 total extracts) before adding DSS to the samples, or to use specific RNA purification methods to remove possible salt contaminants. All the experimental steps are schematized in Figure 3.

## Concluding remarks

Here we provide a straightforward protocol, with comments and tips, which optimizes the classical endogenous RNA PD approach. We show that, at specific concentrations, the addition of DSS during precipitation dramatically increases the recovery of targeted RNAs without affecting their binding to the physiological partners. As an example, we tested the performance of the new set-up on lncMN2-203, whose isolation from 1 mg of DSS-treated extracts was found enhanced. Moreover, we found that the DSS treatment also improved the recovery of miR-466i-5p, previously identified as a functional lncMN2-203 partner (Carvelli et al., 2022). In line with the binding specificity, only background enrichments were found for miR-669a-5p and miR-325-3p, previously shown as non-interacting miRNAs.

Although the RNA PD experiments must be adapted to the specific abundance of the target RNA molecule in the suitable model system, we believe that the improvement here presented will be of great help for many scientists working across the field of RNA biology and networks. Despite the proven effectiveness of the current protocols, the RNA purification still represents a technical challenge for the isolation of classes of transcripts, as lncRNAs, whose expression can be low or diluted across heterogeneous cell populations. This is also highlighted by the recent single cell (sc)RNA sequencing technologies showing that the expression of given RNAs can be extremely cell type specific, thus underrated in total extract, especially for those whose expression is further restricted to specific time and space windows (Savulescu et al., 2020).

## Forthcoming perspectives

We have tested the feasibility and the outcomes of a new RNA precipitation protocol, by narrowing our attention to lncMN2-203, a lncRNA recently demonstrated to be functionally relevant through a mechanism of lncRNA/miRNA interaction. In principle, we cannot predict any technical restriction that may impede, in the future, downstream transcriptomics for a deeper and unbiased identification of the lncRNA-RNA interactome. Moreover, we believe that the improvements to the endogenous RNA PD protocol herein presented could also be applied to future studies on other RNA partners (e.g. proteins) or other RNA substrates. For instance, it may serve as a beneficial tool for the analysis of difficult-to-precipitate ncRNAs, such as circular RNAs (circRNAs), covalently closed lncRNAs generated by back-splicing of canonical pre-mRNAs (Kristensen et al., 2019). The mechanism of action of this class of transcripts often implies the association with other RNAs (Hansen et al., 2013; Piwecka et al., 2017), whose identification is hampered by the fact that the back-splicing junction is the only circRNA distinctive site, as compared to their linear precursors. Additional physical-chemical treatments of the cells (e.g., ultraviolet light or psoralen crosslinking) that have not been tested in the current study, can also be envisaged for the detection of more direct RNA-RNA interactions.

## Data Availability

The original contributions presented in the study are included in the article/Supplementary Material, further inquiries can be directed to the corresponding author.

## References

[B1] Ballarino M. Cipriano A. Tita R. Santini T. Desideri F. Morlando M. (2018). Deficiency in the nuclear long noncoding RNA Charme causes myogenic defects and heart remodeling in mice. EMBO J. 37 (18), e99697. 10.15252/embj.201899697 30177572 PMC6138438

[B2] Bellucci M. Agostini F. Masin M. Tartaglia G. G. (2011). Predicting protein associations with long noncoding RNAs. Nat. Methods 8 (6), 444–445. 10.1038/nmeth.1611 21623348

[B3] Biscarini S. Capauto D. Peruzzi G. Lu L. Colantoni A. Santini T. (2018). Characterization of the lncRNA transcriptome in mESC-derived motor neurons: Implications for FUS-ALS. Stem Cell Res. 27, 172–179. 10.1016/j.scr.2018.01.037 29449089

[B4] Cai Z. Cao C. Ji L. Ye R. Wang D. Xia C. (2020). RIC-seq for global *in situ* profiling of RNA-RNA spatial interactions. Nature 582 (7812), 432–437. 10.1038/s41586-020-2249-1 32499643

[B5] Capauto D. Colantoni A. Lu L. Santini T. Peruzzi G. Biscarini S. (2018). A regulatory circuitry between Gria2, miR-409, and miR-495 is affected by ALS FUS mutation in ESC-derived motor neurons. Mol. Neurobiol. 55 (10), 7635–7651. 10.1007/s12035-018-0884-4 29430619 PMC6132778

[B6] Carrieri C. Cimatti L. Biagioli M. Beugnet A. Zucchelli S. Fedele S. (2012). Long non-coding antisense RNA controls Uchl1 translation through an embedded SINEB2 repeat. Nature 491 (7424), 454–457. 10.1038/nature11508 23064229

[B7] Carvelli A. Setti A. Desideri F. Galfrè S. G. Biscarini S. Santini T. (2022). A multifunctional locus controls motor neuron differentiation through short and long noncoding RNAs. EMBO J. 41 (13), e108918. 10.15252/embj.2021108918 35698802 PMC9251839

[B8] Cipriano A. Ballarino M. (2018). The ever-evolving concept of the gene: The use of RNA/protein experimental techniques to understand genome functions. Front. Mol. Biosci. 5 (20). 10.3389/fmolb.2018.00020 PMC5845540 29560353

[B9] D'Ambra E. Santini T. Vitiello E. D'Uva S. Silenzi V. Morlando M. (2021). Circ-Hdgfrp3 shuttles along neurites and is trapped in aggregates formed by ALS-associated mutant FUS. iScience 24 (12), 103504. 10.1016/j.isci.2021.103504 34934923 PMC8661529

[B10] Desideri F. Cipriano A. Petrezselyova S. Buonaiuto G. Santini T. Kasparek P. (2020). Intronic determinants coordinate charme lncRNA nuclear activity through the interaction with MATR3 and PTBP1. Cell Rep. 33 (12), 108548. 10.1016/j.celrep.2020.108548 33357424 PMC7773549

[B11] Engreitz J. M. Sirokman K. McDonel P. Shishkin A. A. Surka C. Russell P. (2014). RNA-RNA interactions enable specific targeting of noncoding RNAs to nascent Pre-mRNAs and chromatin sites. Cell 159 (1), 188–199. 10.1016/j.cell.2014.08.018 25259926 PMC4177037

[B12] Fatica A. Bozzoni I. (2014). Long non-coding RNAs: New players in cell differentiation and development. Nat. Rev. Genet. 15 (1), 7–21. 10.1038/nrg3606 24296535

[B13] Fatima R. Akhade V. S. Pal D. Rao S. M. (2015). Long noncoding RNAs in development and cancer: Potential biomarkers and therapeutic targets. Mol. Cell. Ther. 3, 5. 10.1186/s40591-015-0042-6 26082843 PMC4469312

[B14] Ferrè F. Colantoni A. Helmer-Citterich M. (2016). Revealing protein-lncRNA interaction. Brief. Bioinform. 1, 106–116. 10.1093/bib/bbv031 PMC4719072 26041786

[B15] Fukunaga T. Hamada M. (2017). RIblast: An ultrafast RNA-RNA interaction prediction system based on a seed-and-extension approach. Bioinformatics 33 (17), 2666–2674. 10.1093/bioinformatics/btx287 28459942 PMC5860064

[B16] Fukunaga T. Iwakiri J. Ono Y. Hamada M. (2019). LncRRIsearch: A web server for lncRNA-RNA interaction prediction integrated with tissue-specific expression and subcellular localization data. Front. Genet. 10, 462. 10.3389/fgene.2019.00462 31191601 PMC6546843

[B17] Goff L. A. Groff A. F. Sauvageau M. Trayes-Gibson Z. Sanchez-Gomez D. B. Morse M. (2015). Spatiotemporal expression and transcriptional perturbations by long noncoding RNAs in the mouse brain. Proc. Natl. Acad. Sci. U. S. A. 112 (22), 6855–6862. 10.1073/pnas.1411263112 26034286 PMC4460505

[B18] Gong C. Maquat L. E. (2011). lncRNAs transactivate STAU1-mediated mRNA decay by duplexing with 3' UTRs via Alu elements. Nature 470 (7333), 284–288. 10.1038/nature09701 21307942 PMC3073508

[B19] Gong J. Shao D. Xu K. Lu Z. Lu Z. J. Yang Y. T. (2018). RISE: A database of RNA interactome from sequencing experiments. Nucleic Acids Res. 46 (D1), D194-D201–D201. 10.1093/nar/gkx864 29040625 PMC5753368

[B20] Guil S. Esteller M. (2015). RNA-RNA interactions in gene regulation: The coding and noncoding players. Trends biochem. Sci. 40 (5), 248–256. 10.1016/j.tibs.2015.03.001 25818326

[B21] Hafner M. Katsantoni M. Köster T. Marks J. Mukherjee J. Staiger D. (2021). CLIP and complementary methods. Nat. Rev. Methods Prim. 1 (1), 20–23. 10.1038/s43586-021-00018-1

[B22] Hansen T. B. Jensen T. I. Clausen B. H. Bramsen J. B. Finsen B. Damgaard C. K. (2013). Natural RNA circles function as efficient microRNA sponges. Nature 495 (7441), 384–388. 10.1038/nature11993 23446346

[B23] Helwak A. Kudla G. Dudnakova T. Tollervey D. (2013). Mapping the human miRNA interactome by CLASH reveals frequent noncanonical binding. Cell 153 (3), 654–665. 10.1016/j.cell.2013.03.043 23622248 PMC3650559

[B24] Herman A. B. Tsitsipatis D. Gorospe M. (2022). Integrated lncRNA function upon genomic and epigenomic regulation. Mol. Cell 82 (12), 2252–2266. 10.1016/j.molcel.2022.05.027 35714586 PMC9219586

[B25] Juritsch A. F. Moreau R. (2019). Rapid removal of dextran sulfate sodium from tissue RNA preparations for measurement of inflammation biomarkers. Anal. Biochem. 579, 18–24. 10.1016/j.ab.2019.05.011 31112717

[B26] Kerr T. A. Ciorba M. A. Matsumoto H. Davis V. R. Luo J. Kennedy S. (2012). Dextran sodium sulfate inhibition of real-time polymerase chain reaction amplification: A poly-A purification solution. Inflamm. Bowel Dis. 18 (2), 344–348. 10.1002/ibd.21763 21618356 PMC3600644

[B27] Kopp F. Mendell J. T. (2018). Functional classification and experimental dissection of long noncoding RNAs. Cell 172 (3), 393–407. 10.1016/j.cell.2018.01.011 29373828 PMC5978744

[B28] Kretz M. Siprashvili Z. Chu C. Webster D. E. Zehnder A. Qu K. (2013). Control of somatic tissue differentiation by the long non-coding RNA TINCR. Nature 493 (7431), 231–235. 10.1038/nature11661 23201690 PMC3674581

[B29] Kristensen L. S. Andersen M. S. Stagsted L. Ebbesen K. K. Hansen T. B. Kjems J. (2019). The biogenesis, biology and characterization of circular RNAs. Nat. Rev. Genet. 20 (11), 675–691. 10.1038/s41576-019-0158-7 31395983

[B30] Lederman L. L. Kawasaki E. S. Szabo P. (1981). The rate of nucleic acid annealing to cytological preparations is increased in the presence of dextran sulfate. Anal. Biochem. 117 (1), 158–163. 10.1016/0003-2697(81)90705-3 6172056

[B31] Lee F. C. Ule J. (2018). Advances in CLIP technologies for studies of protein-RNA interactions. Mol. Cell 69 (3), 354–369. 10.1016/j.molcel.2018.01.005 29395060

[B32] Lekka E. Hall J. (2018). Noncoding RNAs in disease. FEBS Lett. 592 (17), 2884–2900. 10.1002/1873-3468.13182 29972883 PMC6174949

[B33] Lerner M. R. Steitz J. A. (1979). Antibodies to small nuclear RNAs complexed with proteins are produced by patients with systemic lupus erythematosus. Proc. Natl. Acad. Sci. U. S. A. 76 (11), 5495–5499. 10.1073/pnas.76.11.5495 316537 PMC411675

[B34] Li L. Chang H. Y. (2014). Physiological roles of long noncoding RNAs: Insight from knockout mice. Trends Cell Biol. 24 (10), 594–602. 10.1016/j.tcb.2014.06.003 25022466 PMC4177945

[B35] Martone J. Mariani D. Santini T. Setti A. Shamloo S. Colantoni A. (2020). SMaRT lncRNA controls translation of a G-quadruplex-containing mRNA antagonizing the DHX36 helicase. EMBO Rep. 21 (6), e49942. 10.15252/embr.201949942 32337838 PMC7271651

[B36] Matthews B. (1988). Protein-DNA interaction. No code for recognition. Nature 335, 294–295. 10.1038/335294a0 3419498

[B37] McHugh C. A. Chen C. K. Chow A. Surka C. F. Tran C. McDonel P. (2015). The Xist lncRNA interacts directly with SHARP to silence transcription through HDAC3. Nature 521 (7551), 232–236. 10.1038/nature14443 25915022 PMC4516396

[B38] Moore M. J. (2005). From birth to death: The complex lives of eukaryotic mRNAs. Science 309 (5740), 1514–1518. 10.1126/science.1111443 16141059

[B39] Ni Y. Q. Xu H. Liu Y. S. (2022). Roles of long non-coding RNAs in the development of aging-related neurodegenerative diseases. Front. Mol. Neurosci. 15, 844193. 10.3389/fnmol.2022.844193 35359573 PMC8964039

[B40] Phizicky E. M. Fields S. (1995). Protein-protein interactions: Methods for detection and analysis. Microbiol. Rev. 59 (1), 94–123. 10.1128/mr.59.1.94-123.1995 7708014 PMC239356

[B41] Piwecka M. Glažar P. Hernandez-Miranda L. R. Memczak S. Wolf S. A. Rybak-Wolf A. (2017). Loss of a mammalian circular RNA locus causes miRNA deregulation and affects brain function. Science 357 (6357), eaam8526. 10.1126/science.aam8526 28798046

[B42] Ramanathan M. Porter D. F. Khavari P. A. (2019). Methods to study RNA–protein interactions. Nat. Methods 16, 225–234. 10.1038/s41592-019-0330-1 30804549 PMC6692137

[B43] Santini T. Martone J. Ballarino M. (2021). Visualization of nuclear and cytoplasmic long noncoding RNAs at single-cell level by RNA-FISH. Methods Mol. Biol. 2157, 251–280. 10.1007/978-1-0716-0664-3_15 32820409

[B44] Savulescu A. F. Jacobs C. Negishi Y. Davignon L. Mhlanga M. M. (2020). Pinpointing cell identity in time and space. Front. Mol. Biosci. 7, 209. 10.3389/fmolb.2020.00209 32923457 PMC7456825

[B45] Singer R. H. Ward D. C. (1982). Actin gene expression visualized in chicken muscle tissue culture by using *in situ* hybridization with a biotinated nucleotide analog. Proc. Natl. Acad. Sci. U. S. A. 79 (23), 7331–7335. 10.1073/pnas.79.23.7331 6961411 PMC347333

[B46] Singh L. Jones K. W. (1984). The use of heparin as a simple cost-effective means of controlling background in nucleic acid hybridization procedures. Nucleic Acids Res. 12 (14), 5627–5638. 10.1093/nar/12.14.5627 6087294 PMC320019

[B47] Statello L. Guo C. J. Chen L. L. Huarte M. (2021). Gene regulation by long non-coding RNAs and its biological functions. Nat. Rev. Mol. Cell Biol. 22 (2), 96–118. 10.1038/s41580-020-00315-9 33353982 PMC7754182

[B48] Ule J. Jensen K. B. Ruggiu M. Mele A. Ule A. Darnell R. B. (2003). CLIP identifies Nova-regulated RNA networks in the brain. Science 302 (5648), 1212–1215. 10.1126/science.1090095 14615540

[B49] Van Gijlswijk R. P. Wiegant J. Raap A. K. Tanke H. J. (1996). Improved localization of fluorescent tyramides for fluorescence *in situ* hybridization using dextran sulfate and polyvinyl alcohol. J. Histochem. Cytochem. 44 (4), 389–392. 10.1177/44.4.8601698 8601698

[B50] Viennois E. Chen F. Laroui H. Baker M. T. Merlin D. (2013). Dextran sodium sulfate inhibits the activities of both polymerase and reverse transcriptase: Lithium chloride purification, a rapid and efficient technique to purify RNA. BMC Res. Notes 6, 360. 10.1186/1756-0500-6-360 24010775 PMC3847706

[B51] Wahl G. M. Stern M. Stark G. R. (1979). Efficient transfer of large DNA fragments from agarose gels to diazobenzyloxymethyl-paper and rapid hybridization by using dextran sulfate. Proc. Natl. Acad. Sci. U. S. A. 76 (8), 3683–3687. 10.1073/pnas.76.8.3683 291033 PMC383897

[B52] Wetmur J. G. (1975). Acceleration of DNA renaturation rates. Biopolymers 14, 2517–2524. 10.1002/bip.1975.360141208 34098662

[B53] Wichterle H. Lieberam I. Porter J. A. Jessell T. M. (2002). Directed differentiation of embryonic stem cells into motor neurons. Cell 110, 385–397. 10.1016/s0092-8674(02)00835-8 12176325

[B54] Wichterle H. Peljto M. (2008). Differentiation of mouse embryonic stem cells to spinal motor neurons. Curr. Protoc. Stem Cell Biol. Chapter 1, Unit 1H.1.1–1H.1.9. 10.1002/9780470151808.sc01h01s5 18770634

[B55] Yao R. W. Wang Y. Chen L. L. (2019). Cellular functions of long noncoding RNAs. Nat. Cell Biol. 21, 542–551. 10.1038/s41556-019-0311-8 31048766

