# Supplementary figures and images for: Advances in endogenous RNA pull-down: A straightforward dextran sulfate-based method enhancing RNA recovery
Source: Front Mol Biosci. 2022 Oct 19;9:1004746. doi: 10.3389/fmolb.2022.1004746 (PMC9629853; doi:10.3389/fmolb.2022.1004746)

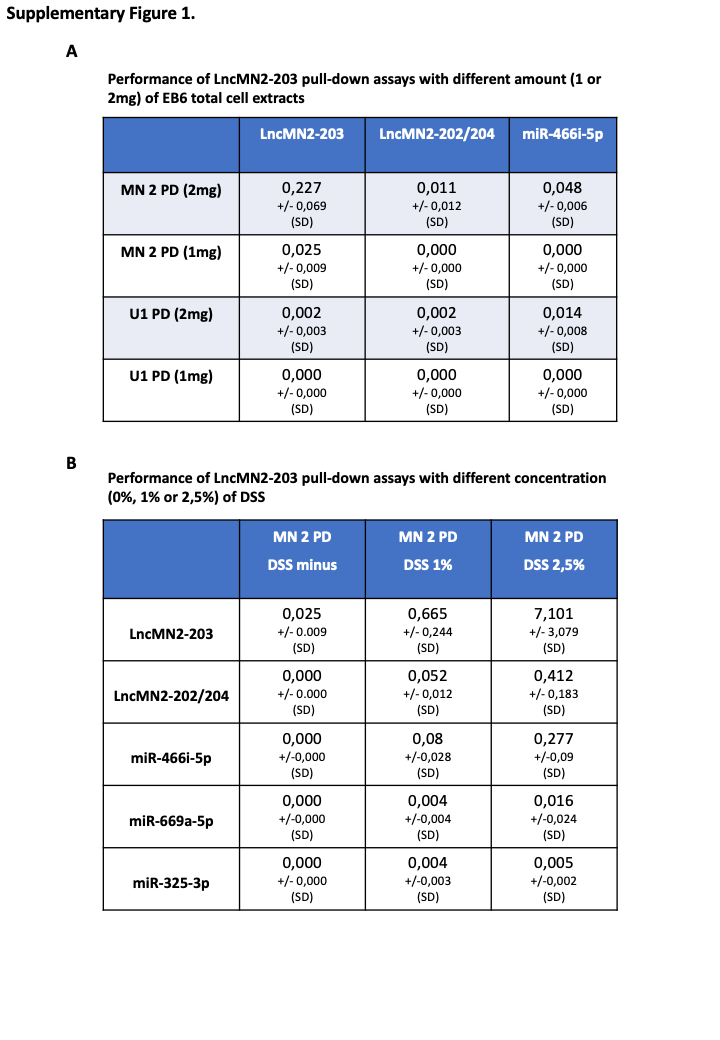

Supplement: Supplementary file 1 [file Image1.tiff]
